# Supplementary material for: Multiple scattering dynamics of fermions at an isolated p-wave resonance
Source: Nat Commun. 2016 Jul 11;7:12069. doi: 10.1038/ncomms12069 (PMC4942570; doi:10.1038/ncomms12069)
Supplement: Supplementary Information — Supplementary Figures 1-2, Supplementary Notes 1-3 and Supplementary References. [file ncomms12069-s1.pdf]

Supplementary Figures

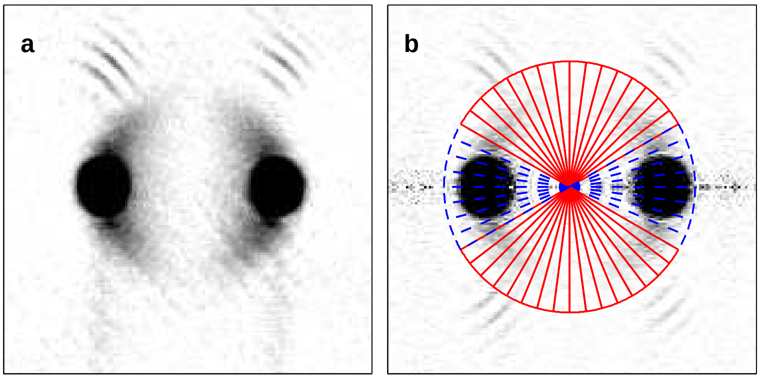

Supplementary Figure 1 | Transformation and binning of absorption data. (a) Absorption data for a collision at $E/k = 150\ \mu$K. **(b)** Image after Abel inversion with 48 angular bins drawn in red and blue. Red solid bins are included in the determination of the scattering distribution, whereas the blue dashed bins are excluded.

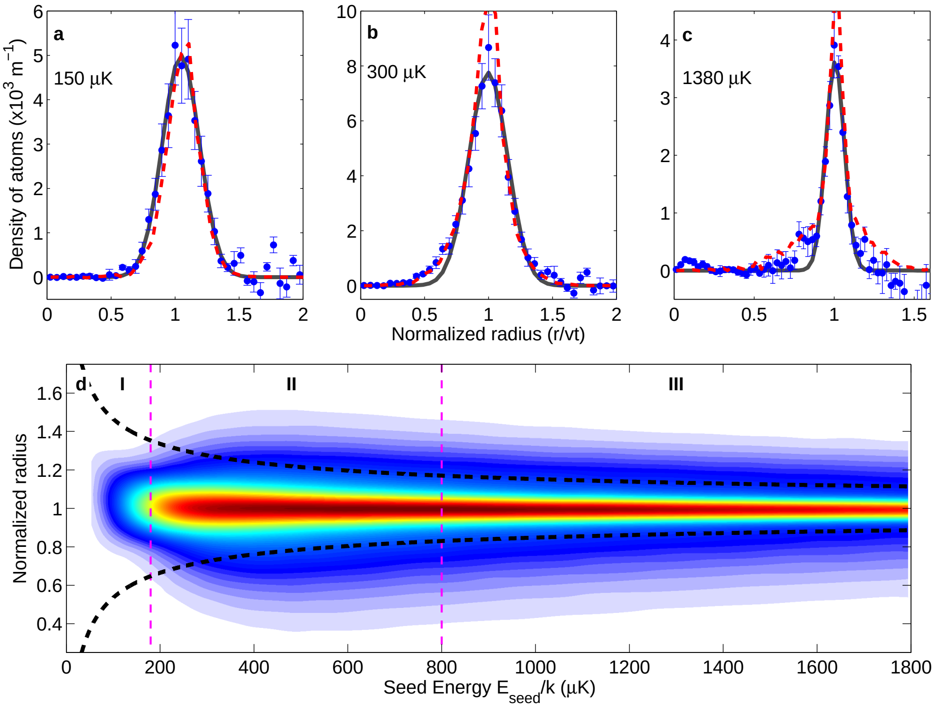

Supplementary Figure 2 | Radial distribution of scattered atoms. (a-c), Blue circles are the mean number of atoms in a given radial bin, averaged over five experimental images. Uncertainties are a combination of statistical errors (one standard deviation) and a 4% systematic error in determining the atom number. The radial bins are normalized as the ratio of measured position to vt, with v the speed and t the time-of-flight. The grey solid line is a fit of the experimental data to a Gaussian. The red dashed line is the DSMC simulation result. **(d)**, Color plot is the distribution of normalized radial positions as a function of nominal collision energies, extracted from the simulated data. Black dashed lines indicate indicate thrice the expected standard deviation of Supplementary Equation (4), normalized to the mean value. Pink dashed lines delineate the three collision regions.

Supplementary Note 1: Image processing

The scattering distribution from each experimental run is determined from a set of three images. The first image is that of a resonant optical probe field after it has passed through the atoms. The second is of the same field but without the atoms present. The third image is without the probe field present in order to measure ambient light levels and the bias of camera. The optical depth, given as $OD = \int_{-\infty}^{\infty} n(x, y, z)\sigma dx = \tilde{n}(y, z)\sigma_{\mathrm{abs}}$, can be determined from the three intensity images I_{atoms}, $I_{\mathrm{no\ atoms}}$, and I_{dark} as

$$OD = -\log\left(\frac{I_{\mathrm{atoms}} - I_{\mathrm{dark}}}{I_{\mathrm{no\ atoms}} - I_{\mathrm{dark}}}\right) + \left(\frac{I_{\mathrm{no\ atoms}} - I_{\mathrm{atoms}}}{I^*_{\mathrm{sat}}}\right), \tag{1}$$

where I^*_{sat} is the effective saturation intensity corresponding to our imaging polarization[1]. Corrections to Supplementary Equation (1) for off-resonant light (leading to a maximum OD) can be ignored in the limit of low optical depth. In principle, the method of Supplementary Equation (1) should eliminate the effect of fringes caused by interference effects in the vacuum cell or dust, but in practice the finite amount of time between acquiring I_{atoms} and $I_{\mathrm{no\ atoms}}$ leads to a finite extinction of these fringes. To further reduce these effects, we use a fringe-removal method[2] that matches (in a least-squares sense) I_{atoms} to a reference image $I_{\mathrm{no\ atoms}}$ taken from a set of such images. We determine the integrated number density $\tilde{n}(y, z)$ by dividing the optical depth by the absorption cross-section $\sigma_{\mathrm{abs}} = \frac{3\lambda^2}{2\pi\alpha^*}$ with wavelength $\lambda = 766.7$ nm and polarization correction factor $\alpha^* = 1.98 \pm 0.10$. We determine α^* by imaging expanded clouds over a wide range of saturation parameters[1], and our measurement agrees well with the expected value for linear $(\sigma^+ + \sigma^-)$ probe light in the absence of a magnetic field of $55/28 \approx 1.96$.

The absorption image (Supplementary Fig. 1a) does not directly yield the scattering distribution; rather, the image is a projection of the 3D scattering distribution onto a 2D plane. As the scattering distribution is cylindrically symmetric the projection is effectively an Abel transform, and we use the BASEX method[3] to transform the image from (y, z)-space to (r, z)-space. While the original paper assumed mirror symmetry in z, we relax this requirement as the initial densities of the original atom clouds are not balanced. The BASEX method has the drawback of introducing noise along the $r = 0$ line, but as our scattering halo is far from $r = 0$ this noise does not affect the analysis.

We bin the Abel-inverted image into angular segments to extract the angular scattering distribution as shown in Supplementary Fig. 1b. First, we determine the locations of the unscattered clouds from fits to Gaussian functions, and these parameters determine the centre of the scattering halo and its approximate radius. We then draw a circle around the halo with a centre coincident with that of the halo and a radius given by the halo radius plus several times the z-width of the unscattered clouds so that we count all participating atoms. This circle is then divided angular sections of width $d\theta = 2\pi/N$, with N between 48 and 96, although the precise value is unimportant. We estimate the angular size of the unscattered clouds by their widths in the y-direction and their z location, and exclude those bins from the analysis. Within each angular bin, we sum over all pixels in that bin to determine the number of atoms $dN_{\mathrm{sc}}(\theta)$ and calculate the angular scattering distribution as

$$\frac{dN_{\mathrm{sc}}}{d\Omega}(\theta) = \frac{dN_{\mathrm{sc}}(\theta)}{\sin\theta d\theta}. \tag{2}$$

The angular scattering distribution is proportional to the differential cross-section, with the constant of proportionality depending on the details of the initial cloud shapes and densities, as well as on the total scattering cross-section.

To determine the total number of scattered atoms we fit $dN_{\mathrm{sc}}/d\Omega$ to the function $D(\theta) = a\cos^2\theta + b$ and integrate $D(\theta)$ over all angles, giving $N_{\mathrm{sc}} = \frac{4\pi}{3}a + 4\pi b$. We use the fit to calculate N_{sc} rather than directly integrating $dN_{\mathrm{sc}}/d\Omega$ as it allows us to interpolate the angular distribution over the excluded angles that are contaminated by the dense, unscattered clouds.

Supplementary Note 2: Global fit

The total number of atoms participating in each collision is a crucial parameter in comparing the DSMC simulation to the experimental data. Unfortunately, we cannot measure the total number of atoms present in each image as the optical depth of the unscattered clouds is far beyond the saturation optical depth of our imaging system. A longer time of flight could solve this issue; however, when there are few scattered atoms the signal-to-noise in determining the scattering distributions is too low to be reliable. Furthermore, at high energies the imaging field of view is too small to allow sufficient expansion. Instead, we fit the simulated curve of $N_{\rm sc}$ to the experimental data by varying the total number of atoms used in the simulation at a fixed temperature of $T = 1.3$ μK, and we find an optimum value at $8.2 \pm 0.15 \times 10^5$ atoms where the uncertainty is determined by the statistical and systematic errors on the experimental $N_{\rm sc}$. Including the uncertainty in the temperature gives a final value of $8.2 \pm 0.6 \times 10^5$. An independent measure of the number of atoms at a long time-of-flight without colliding the two clouds yields $9.6 \pm 1.0 \times 10^5$ atoms. The fitted value for the number of atoms is about 15% less than what we obtain from the reference image, and the discrepancy is likely because atoms escape from the optical trap during the acceleration phase of the collision due to a reduction in trap depth from inertial forces.

There are slight deviations between the the simulated and experimental values of $N_{\rm sc}$ at high and low energies. The deviations at high energy are due to the aforementioned reduction in trap depth which is more pronounced at high accelerations, and hence high energies; given the depth of our trap, the maximum energy which we can access is about 2 mK. The deviations at low energy arise from anisotropic and isotropic contributions. For the anisotropic contribution there are two sources of error: limited signal-to-noise for small numbers of scattered atoms over small angular ranges leads to, on average, overestimates of the parameter a in $D(\theta)$ as fringes and vertical leakage of atoms from the horizontal beam can skew the fitted angular distribution, and more atoms in total participate in the collision as fewer atoms escape from the optical trap for low acceleration (and hence low energy). The isotropic contribution is likely due to a small number of atoms residing in spin states other than $|9/2, 9/2\rangle$ which could then participate in s-wave collisions with the majority spin state. Our imaging is sensitive to only the $F = 9/2$ manifold, and Stern-Gerlach imaging of the $F = 9/2$ manifold revealed no atom states other than $|9/2, 9/2\rangle$; therefore, any possible minority atoms must reside in the $F = 7/2$ manifold. Assuming all minority atoms are scattered and that we do not image the minority atoms, we estimate that the number of minority atoms is approximately 4×10^4 atoms, implying a state purity of 95%.

Supplementary Note 3: Radial distributions

In the case of multiple scattering the radial distribution of scattered atoms also contains useful information. If there are only single scattering events, then the distribution of relative velocities is

$$P(\mathbf{v}_{\rm rel}) = \frac{e^{-(\mathbf{v}_{\rm rel}+v_0\hat{z})^2/2s^2}}{(2\pi s^2)^{3/2}}, \tag{3}$$

where $v_0 = \sqrt{4E/m}$ is the speed corresponding to the relative collision energy E and $s^2 = 2kT/m$. The distribution of the relative speeds $v_{\rm rel} = |\mathbf{v}_{\rm rel}|$, which when combined with the known time-of-flight gives the radial position of the atoms, is then

$$P(v_{\rm rel}) = \frac{1}{v_0\sqrt{2\pi s^2}}\left(e^{-(v_{\rm rel}-v_0)^2/2s^2} - e^{-(v_{\rm rel}+v_0)^2/2s^2}\right). \tag{4}$$

In the limit of high collision energies, where $v_0 \gg s$, Supplementary Equation (4) can be approximated as a single Gaussian distribution with mean v_0 and standard deviation s. Therefore, in analyzing the radial distributions we are most interested in deviations of the measured radial distribution from a Gaussian form.

To generate radial distributions, we use nearly the same method for analyzing the images as for the angular distributions, but instead of binning the image by angle we bin it by radial distance from the collision midpoint. Additionally, we apply a Fourier-based filter to remove strong fringes on the absorption images caused by the scattered light wave from the atoms rebounding from the glass cell. Angles close to the collision axis, where the

unscattered clouds dominate the image, are excluded from the summation. We find that by binning images in both radial and angular coordinates that the radial distribution of atoms is largely independent of angle, thus justifying our choice to sum over all angles. The same image analysis is applied to the synthetic absorption images from DSMC to ensure a fair comparison. The results are shown in Supplementary Fig. 2. Only in region I are the experimental speed distributions well-described by a Gaussian function, indicating that only single scattering events occur in that region. In regions II and III, however, the experimental distributions show significant deviations from the expected Gaussian forms, having more atoms at higher and lower energies than would be expected. These broader distributions are a result of multiple scattering, in correspondence with the results from our DSMC simulations.

Supplementary References

[1] Reinaudi, G., Lahaye, T., Wang, Z. & Guéry-Odelin, D. Strong saturation absorption imaging of dense clouds of ultracold atoms. *Opt. Lett.* **32,** 3143–3145 (2007).

[2] Ockeloen, C. F., Tauschinsky, A. F., Spreeuw, R. J. C. & Whitlock, S. Detection of small atom numbers through image processing. *Phys. Rev. A* **82,** 061606(R) (2010).

[3] Dribinski, V., Ossadtchi, A., Mandelshtam, V. A. & Reisler, H. Reconstruction of Abel-transformable images: The Gaussian basis-set expansion Abel transform method. *Rev. Sci. Instrum.* **73,** 2634–2542 (2002).
